# Climatic Variations as Indicators of Vitamin D Levels and Benign Paroxysmal Positional Vertigo

**DOI:** 10.7759/cureus.18811

**Published:** 2021-10-15

**Authors:** Giorgos Sideris, Marilia C Sapountzi, Vangelis Malamas, George Korres, Alexander Delides, Thomas Nikolopoulos

**Affiliations:** 1 Second Ear-Nose-Throat Department, Attikon University Hospital, National and Kapodistrian University of Athens School of Medicine, Athens, GRC; 2 Department of Infomatics, University of Peiraeus, Peiraeus, GRC

**Keywords:** climate, inner ear, dix hallpike, vestibular disorder, climatic variations, seasonality, vitamin d, benign positional paroxysmal vertigo

## Abstract

Objective

To investigate the possible correlation between benign paroxysmal positional vertigo (BPPV), seasonality, and climatic variations as indicators of vitamin D deficiency, since otoconia are calcium carbonate crystals.

Methods

This is a study of patients who received the diagnosis of BPPV from September 2015 to August 2019. Gender, age, and month of diagnosis were factors recorded and analyzed. The cut-off age of 50 years is used to include osteoporotic patients and postmenopausal women. Meteorological and climatic data of latitude, temperature, sunshine hours, humidity, precipitation, wind force, atmospheric pressure, and horizontal solar irradiance were collected.

Results

Four hundred and eighty-five patients were included in the study; 206 were male (42%) and 279 were female (58%). The mean age was 57.8±15.4 and 54.9±13.9, respectively; 192 patients were ≤50 years old (121 female and 71 male) and 293 patients were over 50 years old (135 male and 158 female). A statistical significance in seasonal variation during autumn months was demonstrated (p-value= 5.2 e-05, z-statistic: 9.8164). There was no statistical correlation between the median number of BPPV patients and the median sunshine hours per month, horizontal solar irradiance, or other climatic variables.

Conclusions

Our study demonstrates seasonality in BPPV patients in Greece but no correlation between BPPV and climatic variations as a proxy for Vitamin-D levels was documented.

## Introduction

Benign paroxysmal positional vertigo (BPPV) is one of the most common peripheral vestibular diseases, characterized by brief episodes of vertigo triggered by abrupt changes in the position of the head. BPPV is caused by the displacement of utricular otoconia into one of the inner ear's three semicircular canals. Although the posterior canal is the most frequently affected, it can also affect the superior and horizontal semicircular canals [1].

According to Minasyan et al., vitamin D receptor deficiency is associated with decreased balance function in mice. This observation leads to the hypothesis that there is a pathogenetic link between human balance control and low vitamin D levels [2]. After all, because otoconia crystals are composed of the aminoacids otoconin and otolin, which are involved in the formation of calcium carbonate, a link between otolithic disorders and vitamin D deficiency is highly likely [3,4]. Vitamin D2 is obtained through diet, while vitamin D3 is produced in the skin when exposed to ultraviolet B (UVB) radiation. Vitamin D produced by the skin or ingested is converted to 1.25-dihydroxyvitamin D, the active form of vitamin D, and released into the circulation. Vitamin D deficiency is typically observed in individuals who do not receive adequate sun exposure, do not consume certain types of vitamin D-rich foods, have impaired nutrient absorption, or are at an increased risk of osteoporosis.

There is still some debate in the published literature about whether seasonality and vitamin D deficiency are real risk factors for BPPV infection [5-9]. Our study will attempt to establish a correlation between the occurrence of BPPV and specific time periods in Greece when vitamin D levels are relatively low in the general population, namely autumn and winter, using climate data as a proxy for serum D levels.

## Materials and methods

This is a retrospective study of patients who presented to the ENT emergency clinic between September 2015 and August 2019 with dizziness and were diagnosed with BPPV. Following a thorough medical history, all patients underwent a comprehensive neurotological examination. The Dix-Hallpike or supine head roll tests were used to establish the diagnosis, while the straight head hanging position was used to raise suspicion of an affected superior semicircular canal. Patients with other peripheral nystagmus-related disorders such as vestibular neuritis, Meniere's disease, or any identifiable pathological signs or symptoms of central nervous system nystagmus or cranial trauma were excluded. Pregnant women and patients who received vitamin D or calcium supplementation at the time of diagnosis were also excluded from the study.

Gender, age, season, and month of diagnosis were all recorded and analyzed as variables. The seasons are defined as spring (March, April, May), summer (June, July, August), autumn (September, October, November), and winter (December, January, February). For the purpose of this report, the population at risk of osteoporosis was considered to include 50 years as a cut-off age in order to include osteoporotic patients and postmenopausal women, as has been done in the majority of other research on this subject [10,11].

All patients were citizens of Athens, Greece (Latitude: 37° 58' 46.02" N, Longitude: 23° 42' 58.39" E). Climatic data of temperature, sunshine hours, humidity, precipitation, wind force, atmospheric pressure, and horizontal solar irradiance were collected by the National Meteorological Service.

Rayleigh test for non-uniformity of circular data was used, for finding the possible seasonal correlation. The parameters are described using seasonal mean and standard deviation. The predictive accuracy was expressed with the 95% confidence intervals (CIs). The level of significance was set to 0.05. The statistical analysis was performed using IBM SPSS Statistics version 19 (IBM Corp., Armonk, NY) and MATLAB, version 9.6, R2019a (MathWorks, Inc., Natick, MA).

## Results

**Figure 1:**
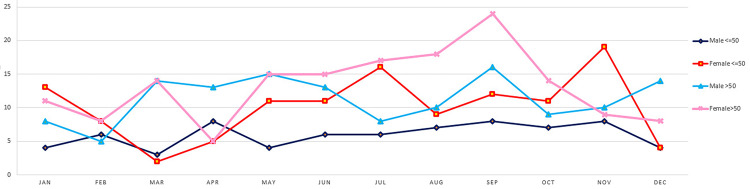
Incidence of BPPV patients by sex and age, with 50 years as cut off BPPV: benign paroxysmal positional vertigo.

Four hundred and eighty-five patients were included in the study with a mean age of 56.1 ± 14.6 years; 206 were male (42%) and 279 were female (58%). The mean age of male and female patients was 57.8±15.4 years (CI: 55.71-59.89) and 54.9±13.9 years (CI: 53.28-56.52), respectively, with no statistical significance. After subgrouping patients according to their age, 192 were under 50 years (121 females and 71 males) and 293 were over 50 years (135 males and 158 females; Figure 1).

The results of the patients' number per month and season are indicating rising incidence in the autumn seasons (Table 1).

**Table 1:** BPPV patients per month and season BPPV: benign paroxysmal positional vertigo

	Patients per month			Female		Male		Total	
	Female (n=279)	Male (n=206)	Total (n=485)	Mean per month	Mean per season	Mean per month	Mean per season	Per month	Per season (%)
Jan	24	12	36	6	13	3	10.25	9	23.25
Feb	16	11	27	4		2.75		6.75	
Mar	16	17	33	4	13	4.25	14.25	8.25	27.25
Apr	10	21	31	2.5		5.25		7.75	
May	26	19	45	6.5		4.75		11.25	
Jun	26	19	45	6.5	21.5	4.75	12.5	11.25	34
Jul	33	14	47	8.25		3.5		11.75	
Aug	27	17	44	6.75		4.25		11	
Sep	36	24	60	9	22.25	6	14.5	15	36.75
Oct	25	16	41	6.25		4		10.25	
Nov	28	18	46	7		4.5		11.5	
Dec	12	18	30	3		4.5		7.5	

**Figure 2:**
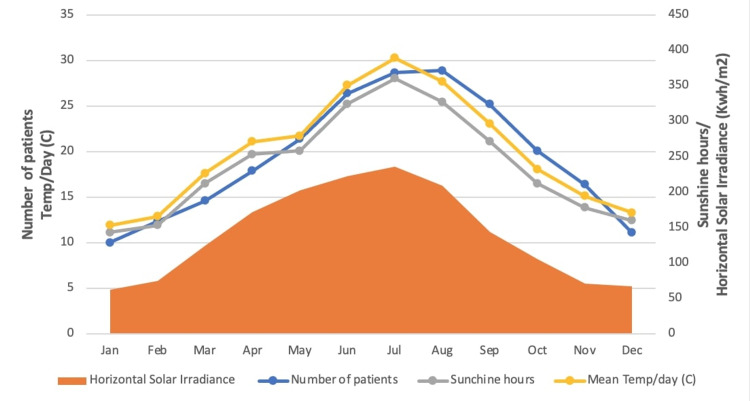
Comparison of monthly BPPV patient numbers, daily sunlight hours, and horizontal solar irradiance BPPV: benign paroxysmal positional vertigo

**Figure 3:**
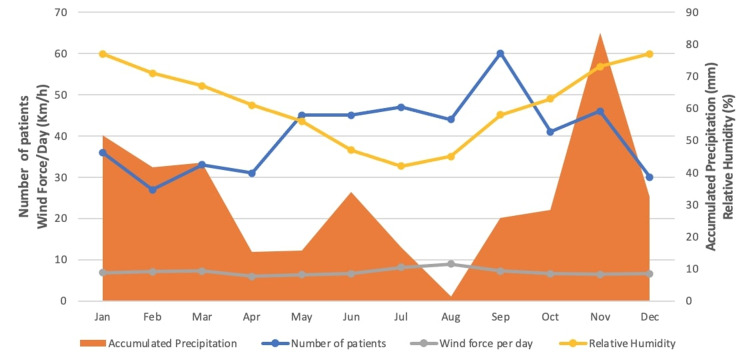
Means of climatic variations in Athens, Greece, from September 2015 to August 2019

The median number of BPPV patients, median sunshine hours per month, horizontal solar irradiance, and other climatic variations are described and demonstrate no statistical significance between the month of BPPV onset and months with low serum Vitamin D levels (Figures 2 and 3).

## Discussion

Calcium homeostasis is critical for the formation and absorption of otoconia. Numerous authors establish a link between BPPV and low vitamin D levels by measuring serum 25-hydroxyvitamin D levels or by highlighting osteoporosis and postmenopausal women as risk factors for BPPV [12-14].

Several studies mention the statistically significant difference between the incidence of BPPV in months when Vitamin D levels are lower compared to those months that these levels are higher while others report on specific atmospheric data such as barometric pressure and its possible relationship with BPPV [5-7,15-17]. On the other hand, recent studies report no seasonality in BPPV patients [8,9]. Seidel et al. found no seasonal variation among 11.153 patients with BPPV [18]. Similarly, a meta-analysis conducted by AlGarni et al. failed to establish a correlation between BPPV occurrence and low vitamin D levels [19].

Females account for 58% of the total sample in our study, corroborated by recent literature [1,5,7,16,18]. Despite the female predominance, no statistically significant differences in sex or age subgroups were observed. The observation that 60% of BPPV patients were over 50 years old suggests that additional comorbidities may exist and contribute to the pathogenesis of BPPV. In fact, several authors report migraines, diabetes mellitus, hypertension, and anxiety as bad prognostic factors [20-22].

While BPPV is more prevalent in the autumn months, other significant atmospheric variables associated with adequate vitamin D intakes, such as horizontal solar irradiance, sunshine hours, temperature, humidity, precipitation, and barometric pressure, are not statistically associated with an increased number of BBPV patients. If the scenario "decreased UVB intake=increased BPPV patients" were true, a higher proportion of BPPV patients should be expected during months with low vitamin D levels, such as the winter months. According to our data, 19% of our patients contract BPPV in the winter and 28% in the summer. Given that the synthesis of vitamin D occurs within a few days after exposure to sunlight, this observation appears quite paradoxical given that the mean sunshine hours and solar irradiance are at their peak during the summer months [23]. Additionally, why is there only a seasonal increase in the autumn months that does not last through the winter?

Numerous parameters could account for these findings. To begin, calcium homeostasis is a multifactorial process that is directly regulated by the parathyroid gland, PTH, calcitonin, and calcitriol. Additionally, numerous factors influence the action of otoconia's formation proteins, the most significant of which is Otoconin-90, which interacts with other minor calcium-binding proteins [24]. Guerra and Devesa report that the presence of several endocrinological and metabolic factors, including age, estrogens, thyroid and growth hormones, corticosteroids, and drugs, alters protein interactions and the binding or availability of calcium, thereby altering the otoconical mineralization process [25]. Second, the composition of vitamin D is determined by a variety of other factors (not just exposure to sunlight and UVB radiation), the most significant of which are insufficient vitamin D synthesis (dark skin, age, obesity), geographic factors (latitude), dietary habits, malabsorption syndrome, perinatal factors, genetic or endocrine disorders, and pregnant or postmenopausal women at increased risk for osteoporosis and medications [4]. It is worth noting that Athens' latitude is 37° (latitudes greater than 35° are associated with decreased vitamin D production because the sun's rays do not fall vertically on the earth), the Greek population has dark skin (dark skin has a low vitamin D content because melanin acts as a barrier to radiation), and regional cuisine is based on olive oil, which is low in vitamin D when compared to fat or butter. These variables were omitted from our analysis due to the paucity of information regarding the patient's prior medical history. Third, the authors’ opinion is that BPPV may be caused by an abrupt or repeated exposure to different temperatures, resulting in abnormalities in the autonomic nervous system. Finally, a clinician should consider additional factors such as a patient's social, economic, and psychological profile and their potential impact on the manifestation of BPPV [1,22].

One could argue that because our study was not population-based, the hospital visits do not accurately reflect true BPPV prevalence. In reality, our hospital emergency visits provide an accurate estimate of the incidence of emergency cases in the Athens metropolitan area (which is home to nearly half of the country's population) and are the only institution open four days a week in western Attica (i.e., the larger Athens area and the referral center for eastern Peloponnese as well as many of the Greek islands). Patients include both emergency referrals and walk-ins. While the population of Athens decreases during the summer months due to vacations, the total number of visits remains constant due to the absence of community physicians and an increase in referrals from vacation areas such as the islands. Additionally, the hospital is a part of the national public health system, and emergency services are available to all citizens and visitors year-round, regardless of their insurance coverage.

Limitation

One of the weaknesses of our study is that we did not measure the actual vitamin D levels of patients. This could not be done because such a measurement is a detour from standard treatment and laboratory testing in the emergency setting and the additional cost was not approved by the IRB. This is why we used an indirect estimation of the Vitamin D levels of the population, a practice that is common in the literature [7,16].

## Conclusions

Our study demonstrates seasonality in BPPV patients in Greece but no correlation between BPPV and climatic variations as a proxy for vitamin-D levels was documented. Our findings suggest that the pathogenesis of BPPV is multifactorial. To establish a more robust evidence base for the relationship between BPPV and vitamin D, larger studies with individualized patient characteristics are required.

## References

[1] Bhattacharyya N, Gubbels SP, Schwartz SR (2017). Clinical practice guideline: Benign paroxysmal positional vertigo (update) executive summary. Otolaryngol Head Neck Surg.

[2] Minasyan A, Keisala T, Zou J (2009). Vestibular dysfunction in vitamin D receptor mutant mice. J Steroid Biochem Mol Biol.

[3] Büki B, Ecker M, Jünger H, Lundberg YW (2013). Vitamin D deficiency and benign paroxysmal positioning vertigo. Med Hypotheses.

[4] Chang SW, Lee HC (2019). Vitamin D and health - the missing vitamin in humans. Pediatr Neonatol.

[5] Meghji S, Murphy D, Nunney I, Phillips JS (2017). The seasonal variation of benign paroxysmal positional vertigo. Otol Neurotol.

[6] Whitman GT, Baloh RW (2015). Seasonality of benign paroxysmal positional vertigo. JAMA Otolaryngol Head Neck Surg.

[7] Saeed BM, Omari AF (2016). Climatic variations and benign paroxysmal positional vertigo. J Otol.

[8] Jeong J, Eo TS, Oh J, Shin HA, Chung HJ, Choi HS (2021). Monthly and seasonal variations in benign paroxysmal positional vertigo. J Vestib Res.

[9] Karataş A, Acar Yüceant G, Yüce T, Hacı C, Cebi IT, Salviz M (2017). Association of benign paroxysmal positional vertigo with osteoporosis and vitamin D deficiency: a case controlled study. J Int Adv Otol.

[10] Hernlund E, Svedbom A, Ivergård M (2013). Osteoporosis in the European Union: medical management, epidemiology and economic burden. A report prepared in collaboration with the International Osteoporosis Foundation (IOF) and the European Federation of Pharmaceutical Industry Associations (EFPIA). Arch Osteoporos.

[11] Makras P, Anastasilakis AD, Antypas G (2019). The 2018 Guidelines for the diagnosis and treatment of osteoporosis in Greece. Arch Osteoporos.

[12] Jeong SH, Kim JS, Shin JW (2013). Decreased serum vitamin D in idiopathic benign paroxysmal positional vertigo. J Neurol.

[13] Yamanaka T, Shirota S, Sawai Y, Murai T, Fujita N, Hosoi H (2013). Osteoporosis as a risk factor for the recurrence of benign paroxysmal positional vertigo. Laryngoscope.

[14] Han W, Fan Z, Zhou M (2018). Low 25-hydroxyvitamin D levels in postmenopausal female patients with benign paroxysmal positional vertigo. Acta Otolaryngol.

[15] Cao Z, Zhao X, Ju Y, Chen M, Wang Y (2020). Seasonality and cardio-cerebrovascular risk factors for benign paroxysmal positional vertigo. Front Neurol.

[16] Zuma FC, de Fraga RB, Ramos BF, Cal RV, Mangabeira Albernaz PL (2019). Seasonality and solar radiation variation level in benign paroxysmal positional vertigo. Acta Otolaryngol.

[17] Korpon JR, Sabo RT, Coelho DH (2019). Barometric pressure and the incidence of benign paroxysmal positional vertigo. Am J Otolaryngol.

[18] Seidel DU, Park JJ, Sesterhenn AM, Kostev K (2019). Demographic data and seasonal variation in peripheral vestibular disorders in ENT practices in Germany. J Vestib Res.

[19] AlGarni MA, Mirza AA, Althobaiti AA, Al-Nemari HH, Bakhsh LS (2018). Association of benign paroxysmal positional vertigo with vitamin D deficiency: a systematic review and meta-analysis. Eur Arch Otorhinolaryngol.

[20] Lawson J, Johnson I, Bamiou DE, Newton JL (2005). Benign paroxysmal positional vertigo: clinical characteristics of dizzy patients referred to a Falls and Syncope Unit. QJM.

[21] von Brevern M, Radtke A, Lezius F, Feldmann M, Ziese T, Lempert T, Neuhauser H (2007). Epidemiology of benign paroxysmal positional vertigo: a population based study. J Neurol Neurosurg Psychiatry.

[22] Cohen HS, Kimball KT, Stewart MG (2004). Benign paroxysmal positional vertigo and comorbid conditions. ORL J Otorhinolaryngol Relat Spec.

[23] Wacker M, Holick MF (2013). Sunlight and vitamin D: a global perspective for health. Dermatoendocrinol.

[24] Andrade LR, Lins U, Farina M, Kachar B, Thalmann R (2012). Immunogold TEM of otoconin 90 and otolin - relevance to mineralization of otoconia, and pathogenesis of benign positional vertigo. Hear Res.

[25] Guerra J, Devesa J (2020). Causes and treatment of idiopathic benign paroxysmal positional vertigo based on endocrinological and other metabolic factors. J Otol.

